# Metabolic Regulations of *Smilax china* L. against β-Amyloid Toxicity in *Caenorhabditis elegans*

**DOI:** 10.3390/metabo14010049

**Published:** 2024-01-13

**Authors:** Lili Yan, Yuchan Deng, Yulan Du, Xutong Fang, Xin Fang, Qiang Zhang

**Affiliations:** 1 Shaanxi Key Laboratory of Natural Products & Chemical Biology, College of Chemistry & Pharmacy, Northwest A&F University, Yangling 712100, China; 2 State Key Laboratory of Phytochemistry and Plant Resources in West China, Kunming Institute of Botany, Chinese Academy of Sciences, Kunming 650201, China

**Keywords:** Alzheimer’s disease, *Smilax china* L., β-amyloid, *Caenorhabditis elegans*, metabolomics, neuroprotective

## Abstract

*Smilax china* L. (Chinaroot) is a natural herb that has multiple uses, such as being used to make tea and food. Both its roots and leaves have different uses due to their unique components. In this study, we analyzed the extract of *S. china.* roots using LC-HRMS and evaluated the neuroprotective effects and metabolic regulation of *S. china* on *Caenorhabditis elegans.* Chinaroot extract prolonged the life span of healthy nematodes, delayed the paralysis time of transgenic CL4176, and reduced the level of β-amyloid deposition in transgenic CL2006. The comprehensive analysis of metabolomics and qRT-PCR revealed that Chinaroot extract exerted neuroprotective effects through the valine, leucine and isoleucine degradation and fatty acid degradation pathways. Moreover, we first discovered that the expressions of *T09B4.8*, *ech-7*, and *agxt-1* were linked to the neuroprotective effects of Chinaroot. The material exerted neuroprotective effects by modulating metabolic abnormalities in AD model *C. elegans*. Our study provides a new foundation for the development of functional food properties and functions.

## 1. Introduction

*Smilax china* L. (Sc), a traditional herb, is widely found in Southeast Asia, including China, and is used a functional food in everyday life. Its roots (Chinaroot) are commonly known as “Baqia” in China [1] and can be used to make “Baqia” tea. It is a rich source of starch and also used as an ingredient in winemaking [2,3]. In China, its shoots and leaves are often utilized as vegetables, while in Korea, the leaves are used to enhance the flavor of rice cakes [4]. Modern pharmacological studies have revealed that active ingredients of Sc are mainly saponins, flavonoids, polysaccharides, and amino acids, and its roots have anti-inflammatory, antioxidant, antitumor, anti-obesity, and other pharmacological activities [5]. For instance, flavonoids isolated from Sc ameliorate pelvic inflammation by promoting autophagic cell reprogramming via the NLRP3 inflammation-mediated autophagy pathway [6]. The methanol extract of Sc root has neuroprotective effects against excitatory poisoning and cerebral ischemic injury, as well as Aβ(25–35)-induced cortical nerve injury in rats [7,8]. Sc polyphenols can also be used to prevent obesity and related metabolic diseases in mice [9].

AD is a prevalent neurodegenerative disease associated with aging that greatly impacts brain function. It can cause a gradual decline in cognitive and memory functions and is characterized by β-amyloid (Aβ) deposition and neurofibrillary [10]. With the aging of the population, the increase in the morbidity of AD will bring a substantial economic and health burden to families and society. Donepezil, memantine, and galantamine are the primary drugs used to clinically treat AD, but these drugs can only relieve AD symptoms and cannot slow down or prevent the development of AD [11]. Therefore, more therapeutic drugs and methods are needed. Accumulation of Aβ in AD people may occur before any tau abnormalities. Aβ is a crucial element in the formation of neuroinflammatory plaques, which are believed to be the trigger and cause of AD [12]. Preventing the buildup of Aβ in the brain could be a promising therapy for AD. Since β-secretase and γ-secretase in the nuclear endosomes produce Aβ during sequential protein hydrolysis of amyloid precursor protein (APP), Aβ can serve as a biomarker for AD [13]. Transgenic *C. elegans* CL2006 expresses a 42 amino acid sequence of human Aβ under the control of somatic muscle-specific *unc-54* promoter/enhancer and exhibits progressive paralytic symptoms with an increasing *C. elegans* life span [14]. The strain CL4176 exhibits paralytic symptoms via the temperature-induced expression of Aβ_1–42_ in somatic wall cells [15]. Thus, both strains could be used as pharmaceutical models for exploring AD pathology and ideal animal models for developing therapeutic drugs for AD.

In addition, according to recent studies, finding new functions of natural plants is significantly aided by liquid chromatography–high-resolution mass spectrometry (LC-HRMS). These studies involve the analysis of specific polyphenolic compounds or the non-targeted analysis of a wide range of polyphenols in the samples. The LC-HRMS methodology allows for the identification and quantification of polyphenols, providing valuable information for quality control, authentication, and studying the bioactive properties of these compounds [16]. Sc is a beneficial economic plant for both medicinal and food purposes. However, there are relatively few studies on the mechanisms of neuroprotection and metabolic regulation of Sc extracts in organisms.

Metabolic abnormalities have been associated with neurodegenerative diseases [17]. Alzheimer’s disease (AD) patients are accompanied by alterations in energy and lipid and amino acid metabolism [18]. Additionally, metabolites play a crucial role in cellular function, and any changes in metabolites reflect the outcome of molecular biology [19]. Thus, differential metabolites of potential interest can be screened to uncover biomarkers related to the disease and used to explore potential pathogenic mechanisms [20]. In recent years, the metabolomic analysis of brain tissue and blood from clinical patients with AD has yielded many emerging candidate biomarkers [21]. For example, a metabolomic analysis of metabolites from the serum and brain tissue of β-amyloid (Aβ)-induced AD model mice revealed that *Bifidobacterium breve* treatment can restore Aβ-induced metabolomic disorder related to amino acids [22]. In our previous study, we used a metabolomic analysis to find that vitamin C can affect the tryptophan metabolism pathway in transgenic *C. elegans* [23].

In our screening for neuroprotective herbs, we discovered that the extract of *Smilax china* L. (ScE) played a significant role in the progression of AD and aging. Considering the metabolomic alternation in the aging or AD development process, we therefore investigated the metabolic regulation in vivo and the related mechanisms to the effects of ScE on anti-aging and anti-AD.

## 2. Materials and Methods

### 2.1. Chemicals and Plants Material

*S. china* was purchased from the flagship store of National Pharmacy. A voucher specimen (No. 20210725) was deposited at Shaanxi Key Laboratory of Natural Products & Chemical Biology, College of Chemistry & Pharmacy, Northwest A&F University.

We obtained the necessary materials for our investigation from Solarbio Science & Technology Co., Ltd. (Beijing Solarbio Science & Technology Co., Ltd., Beijing, China). These materials include tryptone, cholesterol, 5-fluoro-2′-deoxyuridine (Fudr), PBS, agar, Triton-100, and β-mercaptoethanol. Levamisole was purchased from Aladdin (Shanghai Aladdin Biochemical Technology Co., Ltd., Shanghai, China), and Thioflavin T was obtained from Macklin (Shanghai Macklin Biochemical Technology Co., Ltd., Shanghai, China).

### 2.2. Preparation of Sc Root n-BuOH Extracts

The powdered tuberous rhizome of Sc (15 g) was refluxed for 1 h with 150 mL 95% methanol 3 times. The crude extract was filtered, and the solvent was evaporated to yield 3.0 g extract. The extract was ultrasonicated with water for 30 min and then extracted separately with petroleum ether (PE), EtOAc, and n-BuOH 3 times. Then, 0.4 g of n-BuOH extract of Sc roots was obtained after concentrating and evaporation via rotary evaporator, and the drug extract rate of Sc was 2.67%. Finally, the ScE was dissolved in methanol (100 μg/mL), and 2-amino-3-(2-bromophenyl) propionic acid (2 μM) was added as an internal standard for qualitative and quantitative analysis on LC-HRMS. ScE was dissolved to 100 mg/mL with dimethyl sulfoxide (DMSO) and diluted to the specified concentration (1.0~100 μg/mL) of the working solution with OP50 solution.

### 2.3. ScE Chemical Components Analysis via LC-HRMS

The n-BuOH extract was prepared into a 0.1 mg/mL MeOH solvent for LC-HRMS analysis, which was performed using a Q Exactive Focus LC-HRMS equipped with a Vanquish UPLC (Thermos Fisher Scientific, Waltham, MA, USA). The components were separated by a ReproSil-Pur Basic C18 column (150 × 2 mm, 3 µm, Dr. Maisch, High Performance LC GmbH, Ammerbuch, Germany). A 5 μL sample was injected, and the column was kept at 30 °C. In the mobile phase, it contained 0.1% AcOH. In the first 2 min, the mobile phase was 2%. The mobile phase increased up to 100% over the next 20 min. Finally, it remained at 100% for an additional 5 min. The mass spectra were acquired according to ddMS2 protocol, with a resolution of 70,000, scanning ions from 70 to 1000 *m/z*. Positive and negative ions were scanned in separated batch. Collision energies were 10, 20, and 40 V. MS raw data were recorded in profile form. The raw data were analyzed in an open-source package MS-Dial (version 4.9, RIKEN Center for Sustainable Resource Science, Yokohama, Japan) to perform ion identification. The resulting network diagram was plotted using an open-source R library of igraph (version 1.6.0, the igraph core team).

### 2.4. C. elegans Maintenance and Treatment

The wild-type *C. elegans* N2 (*Caenorhabditis elegans*) and transgenic *C. elegans* strains CL4176 and CL2006 were obtained from Caenorhabditis Genetics Center (CGC, University of Minnesota, Minneapolis, MN, USA). We grew all strains on *C. elegans* growth media (NGM) coated with *Escherichia coli strain* (OP50). N2 and CL2006 strains grew under 20 °C, while CL4176 grew under 15 °C. For N2 and CL2006, synchronization was achieved by lysing the adult worms using a lysis solution (5% NaClO, 2M NaOH), whereas, for CL4176, synchronization was achieved by first allowing the adult worms to spawn for 4~5 h and then separating the adult worms from the synchronized worm eggs.

### 2.5. C. elegans Toxicity Assay

To assess ScE toxicity, N2 *C. elegans* was cultured to L4 through synchronization treatment. Afterwards, 50 worms per plate were placed on NGM plates that were either control (0.1% DMSO) or ScE (1, 10, 50, and 100 μg/mL). The worms were cultured in a constant temperature chamber at 20 °C. Surviving *C. elegans* specimens were counted every two days for ten days after transferring to new plates. Toxicity tests were performed using Liu’s method, with slight modifications [24], repeated three times.

### 2.6. Body Bends

The synchronized N2 *C. elegans* (L4) specimens were placed in NGM dishes with either 0.1% DMSO or ScE at concentrations of 1, 10, 50, and 100 μg/mL, and the dishes were switched every two days. Healthy *C. elegans* specimens were randomly selected from each group on days 2, 4, 6, and 8 to be transferred to M9 buffer. They were stewed for 1.0 min to adapt to a new culture base before recording the number of body bends over the following 30 s. The *C. elegans* body bends were counted according to a reported method [14]. Each sinusoidal curve of *C. elegans* bending and recovery was recorded as one movement. Twenty *C. elegans* specimens were counted in each treatment, and three replications were performed.

### 2.7. Reproductive Assay and Body Length

Wild-type N2 *C. elegans* was used for reproductive capacity and body length assay [25]. N2 hermaphrodite *C. elegans* specimens synchronized to L4 were randomly assigned to NGM plates coated with control (0.1% DMSO) or ScE (1, 10, 50 and 100 μg/mL) groups. One *C. elegans* was placed on each plate. Pregnant *C. elegans* specimens were transferred to fresh NGM plates every 24 h. NGM plates with eggs were placed in a 20 °C thermostat for cultivation until adult worms no longer produced eggs. We counted all larvae that hatched from eggs. Each experiment was repeated three times.

Synchronized N2 *C. elegans* of L4 was placed on NGM plates coated with control (0.1% DMSO) and ScE-treated groups (1, 10, 50, and 100 μg/mL). A total of 60–70 nematodes were placed per petri dish. The dishes were replaced every two days, and the culture was allowed to grow for up to five days. After being anesthetized with levamisole (5 mM), the *C. elegans* were placed temporarily on agarose slices (2%) and photographed with a somatic fluorescence microscope SMZ25 (Nikon Corp., Tokyo, Japan) at 5× magnification. Finally, they were quantitatively analyzed for body length, using ImageJ (version 1.53t). We used 30 *C. elegans* per treatment group.

### 2.8. Life Span Assay on N2 and CL4176 C. elegans

After synchronization, 60–70 N2 *C. elegans* specimens were grown until the L4 stage and then placed on NGM plates coated with either control (0.1% DMSO) or ScE (1, 10, 50, and 100 μg/mL). The plates were changed every two days, and when the *C. elegans* started to die, the plates were changed daily. The number of worms was counted until all of them died. Each treatment was repeated three times.

To evaluate whether ScE could extend the life span of CL4176 *C. elegans*, we determined the life span of transgenic *C. elegans* at effective concentrations [26]. Eggs of synchronized CL4176 were incubated in control (0.1% DMSO) and 50 μg/mL ScE NGM dishes for 72 h and then transferred to dishes containing Fudr and coated with control and ScE. At the beginning, the dishes were changed every two days; when the *C. elegans* specimens started to die, the dishes were changed every day, and the number was counted until all *C. elegans* specimens were dead. Each experiment was repeated three times.

### 2.9. Paralysis Assay

The paralysis was assayed with CL4176 following a reported method [27]. The eggs of CL4176 after synchronization were placed on an NGM plate of blank (0.1% DMSO) and ScE-treated groups (1, 10, 50, and 100 μg/mL) and incubated at 15 °C for 36 h. Then, the culture temperature increased to 25 °C to induce the expression of Aβ_1–42_ in their body wall muscle. After 12 h of incubation, the *C. elegans* specimens were observed every 2 h until all of them were paralyzed. *C. elegans* specimens were determined to be in a state of paralysis if the they failed to roll over when being touched with a platinum wire loop or if they could only move their head. This assay was repeated three times with at least 80 *C. elegans* specimens per treatment.

### 2.10. Aβ Deposits Observation

Thioflavin T (ThT) staining was carried out on CL2006 *C. elegans* [28]. The synchronized CL2006 eggs were incubated in NGM at 20 °C in 0.1% DMSO and ScE (50 μg/mL) groups, and the dishes were replaced every two days. On day six, around 100 *C. elegans* specimens were preserved in 150 μL of 4% paraformaldehyde/PBS (pH 7.4) at 4 °C for 24 h. The liquid was taken out by spinning the sample, and the worms were made permeable by 150 μL of fresh osmotic solution (1% Triton X-100, 5% β-mercaptoethanol, 125 mM Tris-HCL, pH 7.4) at 37 °C for another 24 h. Then, the supernatant was centrifuged and removed, and 150 μL of 0.125% ThT was added to stain for 3 min. The *C. elegans* was decolorated to colorlessness with 50% ethanol and was resuspended with M9 buffer. A total of 12 μL M9 solution was added dropwise onto the slide and picked worms to the center of M9 buffer. Slices were sealed and photographed with an inverted fluorescence microscope Dmi8 (Leica Microscope, Wetzlar, Germany) at 40× magnification. The experiment also set N2 *C. elegans* as a control group, and all treatment conditions were consistent with CL2006 *C. elegans*. Finally, Aβ deposition was quantified by ImageJ.

### 2.11. Preparation, LC-HRMS Analysis of C. elegans Metabolome, and Data Analysis

Metabolomics studies were performed using a transgenic *C. elegans* CL4176 to carry out an untargeted metabolism analysis [29]. The experiment was set up with three parallel-treated groups: blank control (0.1% DMSO) and two ScE groups (10 μg/mL and 50 μg/mL). The synchronized CL4176 *C. elegans* eggs were incubated in blank and ScE NGM dishes at 15 °C for 36 h. Then, the *C. elegans* specimens were incubated at 25 °C for another 24 h. The samples were collected and washed three times each with M9 buffer and sterile water. They were then quickly frozen with liquid nitrogen and stored in a refrigerator at −80 °C. There were 8 parallel replicates in each group. The dried worm sample was freeze-dried using a vacuum freeze-dryer. The dried worms were extracted with 80% MeOH (200 µL per 1 mg dried worms) containing 10 µM of 2-amino-3-(2-bromophenyl) propionic acid as the internal standard. The extraction was performed using a disruptor (JY92-IIDN Ningbo Scientz Biotechnology Co., Ltd., Ningbo, China). The ultrasonic power of the cell disruptor was set at 25%, and all operations were conducted with ice water. Each sample was then centrifuged at 12,000 rpm for 10 min at 4 °C. The supernatant was analyzed by LC-HRMS.

The machine analysis and data analysis were performed according to the method we reported [30]. In brief, LC-HRMS was analyzed on a Q Extractive Focus instrument (Thermos Fisher Scientific, Waltham, MA, USA). The components in samples were separated on a Hypersil Gold™ aQ C18 Polar column (150 × 2.1 mm, 1.9 μm, Thermos Fisher Scientific, Waltham, MA, USA) under 30 °C. Gradient MeCN (1~100%) with 0.1% CH_3_COOH was used as an elution. Positive- and negative-ion (70~1000 *m*/*z*) data were collected using the ddMS2 protocol under the collision voltages of 10, 20, and 40 V. The open-source tool MS-Dial version 4.90 was used to import the raw data files for the metabolomic analysis. The MS/MS reference database includes our in-house database (594 metabolites), HMDB (5.0) MS/MS spectra, and CFM-ID (version 4.0)-predicted MS/MS spectra. The differential metabolites were filtered and visualized using an R script-incorporated R packages of mixOmics and ggplot2 with |log2(FC)| > 2.0 and FDR-correlated *p* < 0.01.

### 2.12. Real-Time Quantitative PCR (qRT-PCR)

To further investigate the effect of ScE on the gene expression of disturbed metabolic pathways, we used 0.1% DMSO and 50 μg/mL ScE groups with approximately 2000 *C. elegans* in each group. The sample collection method was the same as that of the metabolomic experiment. Primers were designed by Sangon Biotech online primer design software, and the sequences are shown in Supplementary Table S3. To extract total RNA from the samples, AG RNAex Pro RNA Extraction Reagent was used. cDNA was obtained using the Evo-MLV Reverse Transcription Premix Kit. The qRT-PCR was performed using the CFX 96-touch instrument (Bio-Rad Laboratories, Inc., Hercules, CA, USA). The data analysis was carried out using R language. Each treatment group was repeated at least three times. The quantification was performed using the SYBR Green Pro qRT-PCR analysis. β-actin was used as an internal reference for gene expression.

### 2.13. Statistical Analysis

All experiments were performed thrice, and the mean ± standard deviation was calculated. To determine the *p*-value for life span, we utilized Kaplan–Meier Survival Curves and Log Rank Test. A one-way or two-way analysis of variance (ANOVA) was conducted to analyze any statistical differences among the three groups. We used R (version 4.0) scripts for a statistical analysis, where ns indicates no significant difference; and *, **, ***, and **** indicate *p* < 0.05, *p* < 0.01, *p* < 0.001, and *p* < 0.0001, respectively.

## 3. Results

### 3.1. LC-HRMS Analysis of ScE

**Figure 1:**
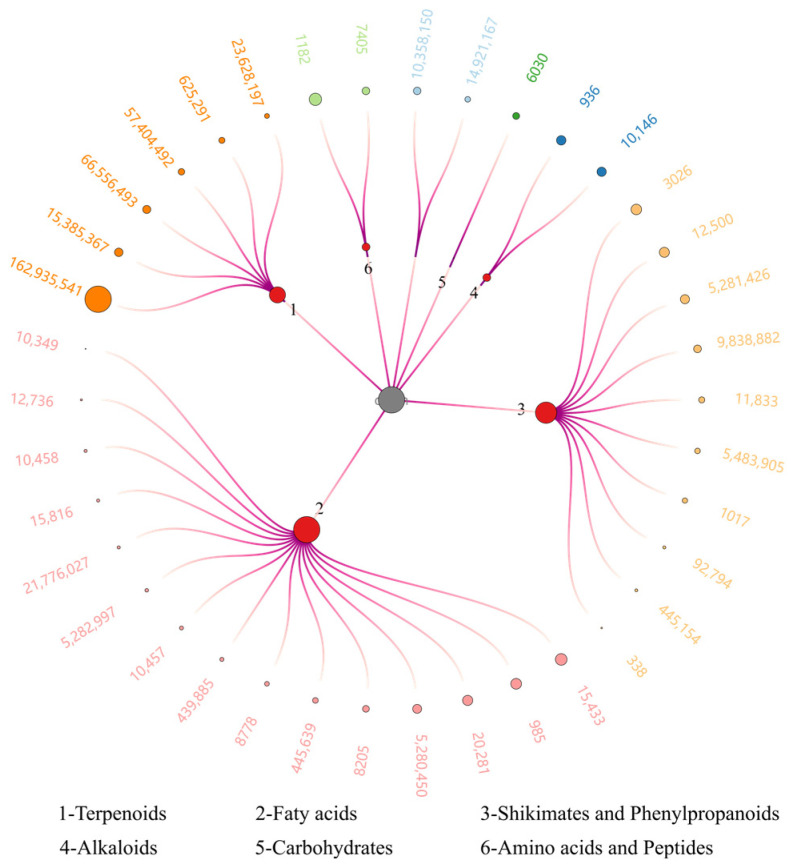
Putatively annotated ScE compounds by LC-HRMS. Size of dots in the outer circle represents relative content according to normalized peak area. Size of dots 1~6 represents the number of child nodes (compounds). For more information, please find the Supplementary Figure S1 and Table S1.

The composition of natural medicines affects their usage. There were multiple reports about the use of methanol and ethanol to extract Sc. These extracts primarily consist of small- and medium-polarity compounds. In our research, we extracted a 95% methanol Sc extract using PE and EtOAc to remove non-polar or low-grade compounds such as fatty acids, aromatic compounds, ketones, and aldehydes. We then used water-saturated n-BuOH to obtain compounds with high polarity. In our subsequent studies, we conducted compositional analysis and activity studies on extracts containing highly polar compounds. The extract of ScE was subjected to LC-HRMS analysis and yielded 38 metabolites categorized into six groups. Terpenoids were the most prevalent macro-polar components (Figure 1 and Supplementary Table S1). The relatively high contained compound identified by LC-HRMS was a terpene, [(1S,2S,6S,7S,11S)-8-(hydroxymethyl)-1,2,6-trimethyl-11-tricyclo [5.3.1.02,6] undec-8-enyl] acetate (PubChem CID 162935541). The fatty acids family had the most prevalent embers, including 15 compounds. Moreover, the found saponins were kaempferol-7-O-neohesperidoside (PubChem CID 5483905) and naringenin-7-O-glucoside (PubChem CID 92794) saponins. However, limited compounds were found due to the limited diverse compounds in our database. The 2D chromatogram and base peak chromatogram of ScE are shown in Supplementary Figure S1.

### 3.2. Effect of ScE on N2 C. elegans Growth Characteristics

**Figure 2:**
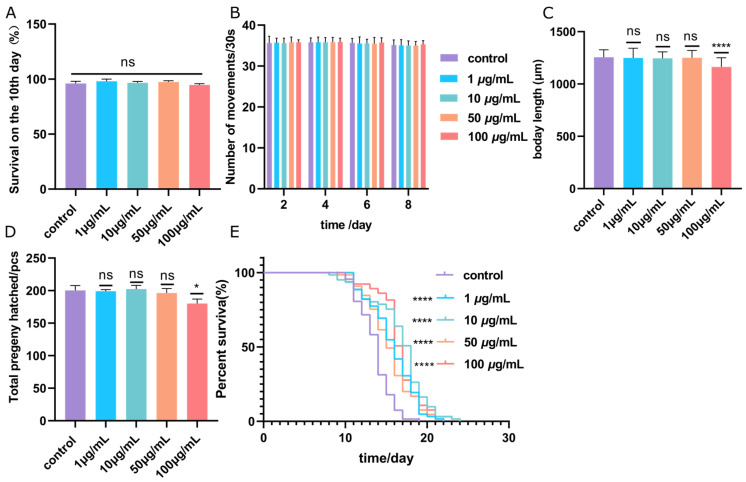
Effect of ScE treatment on *C. elegans* growth indices. (**A**) N2 *C. elegans* survivals during 10-day culture. (**B**) *C. elegans* body length (30 in each duplicate). (**C**) Number of *C. elegans* body movement. (**D**) The total number of progenies hatched of *C. elegans*. (**E**) Life span of *C. elegans* culturing with ScE. *p* < 0.05 (*), *p* < 0.0001 (****), no significance (ns).

To assess the impacts of ScE on *C. elegans* specimens’ growth, we conducted tests on body length, body bends, progeny hatching rate, and life span. The 10-day survival of *C. elegans* was applied to assess the non-toxicity concentration of ScE. It was shown that *C. elegans* safely survived until treated with 100 μg/mL ScE during a 10-day culture (Figure 2A). We then monitored the number of body bends in each group on days 2, 4, 6, and 8. No significant change was found in movement compared to the control group (Figure 2B). However, a minor decrease was observed in body length (Figure 2C and Figure S2) and progeny hatching rate (Figure 2D) at 100 μg/mL ScE exposure. Under the non-toxicity exposure concentrations in 1~100 μg/mL, ScE showed a potential function to prolong the life span of *C. elegans* (Figure 2E). Overall, ScE exhibited a prolonged life span without obvious toxicity.

### 3.3. ScE Alleviated Symptoms of Paralysis of CL4176

**Figure 3:**
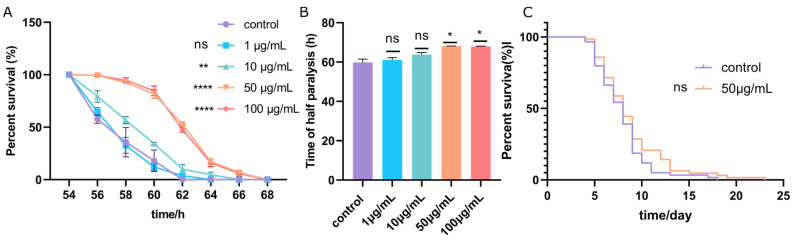
CL4176 *C. elegans* paralysis rate and longevity. (**A**) CL4176 *C. elegans* paralysis rate. (**B**) Time of half paralysis of CL4176. (**C**) Life span of *C. elegans* CL4176. *p* < 0.05 (*), *p* < 0.01 (**), *p* < 0.0001 (****), no significance (ns).

To evaluate the effect of ScE on *C. elegans* paralysis, we evaluated percent survival effected by different concentrations of ScE (1, 10, 50, and 100 μg/mL). As shown (Figure 3A), 1 μg/mL ScE had no effect on delaying *C. elegans* paralysis, but 10, 50, and 100 μg/mL obviously delayed *C. elegans* paralysis. As shown in Figure 3B, half of the *C. elegans* showed delayed paralysis time when the concentration of ScE was 50 and 100 μg/mL. Since the progeny frontal hatching rate, as well as the body length, of *C. elegans* could be negatively affected by 100 μg/mL ScE, we therefore selected 50 μg/mL extract for the subsequent investigations. A total of 50 μg/mL of ScE did not significantly increase the life span of CL4176 compared to the control (Figure 3C), indicating that ScE does not extend the life span of Aβ-induced *C. elegans*.

### 3.4. ScE Reduced Aβ Aggregation in C. elegans CL2006 Strain

**Figure 4:**
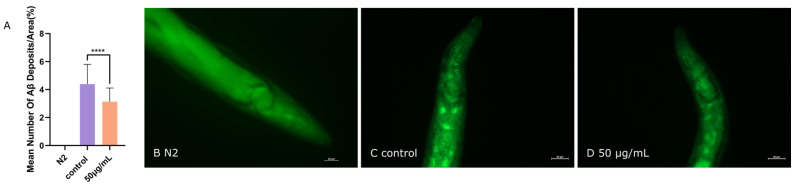
ScE reduced Aβ (1–42) aggregation in CL2006 transgenic worms by ThT staining assay. (**A**) Mean number of Aβ deposits. *p* < 0.0001 (****). (**B**–**D**) Thioflavin T-staining images.

A promising strategy for treating AD is to prevent Aβ aggregation [31]. ThT can be used to visualize and quantify stacked β-amyloid fibrils or Aβ deposition in the head of the *C. elegans* CL2006 strain [32]. In our investigations (Figure 4A), the Aβ deposition was detected in CL2006 (Figure 4C,D) but not in the wild type (Figure 4B). After treatment with 50 μg/mL extract, the average number of Aβ deposits in the *C. elegans* decreased significantly (Figure 4A,C,D). These findings suggest that ScE treatment can reduce Aβ accumulation and protect *C. elegans* from Aβ-induced toxicity.

### 3.5. C. elegans CL4176 Metabolome Interfered by ScE

**Figure 5:**
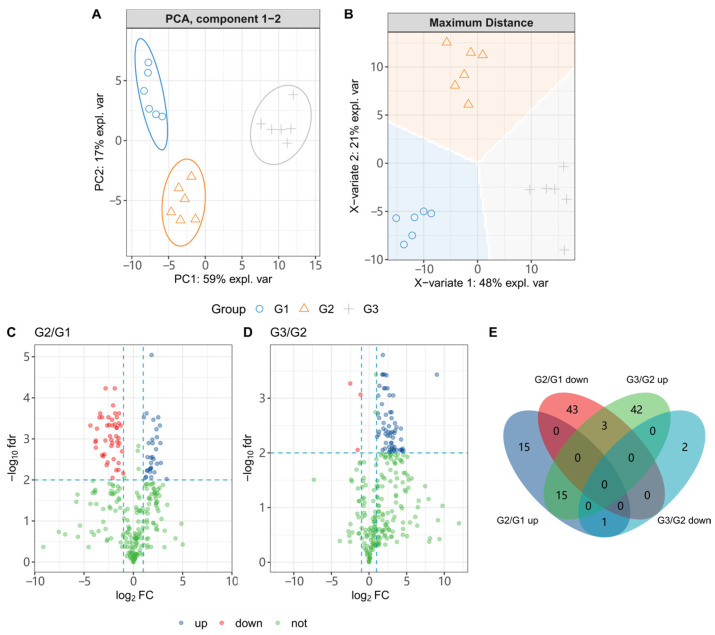
Trends of metabolites in CL4176 *C. elegans*s after G1 (0.1% DMSO), G2 (10 μg/mL), and G3 (50 μg/mL) treatments. (**A**) PCA and (**B**) sPLS-DA analysis (blue “○” represents G1 (0.1% DMSO), yellow “Δ” represents G2 (10 μg/mL), and gray “+” represents G3). Metabolism variation induced by ScE (**C**,**D**). Blue “○” represents upregulated differential metabolites; red “○” represents downregulated differential metabolites, and green “○” represents nonchanged differential metabolites. (**E**) The number of differential metabolites.

We conducted metabolomics studies on CL4176 to determine if ScE could affect *C. elegans* metabolites. To obtain the metabolites, we treated CL4176 with G1 (0.1% DMSO), G2 (10 μg/mL), and G3 (50 μg/mL). Our results, as shown in Figure 5A,B, demonstrate a clear separation of G1, G2, and G3. PCA analysis, an unsupervised method, was used to reflect the grouping and differences of the samples through the dimensionality reduction process. PC1 and PC2 explained 59% and 17% of the differences, respectively. The groups were clearly separated from one another and strongly clustered, which is consistent with the various experimental treatments. Additionally, sPLS-DA (Sparse Partial Least Squares Discriminant Analysis), a supervised modeling method for classifying samples, was used to provide clear categorical features. As shown in Figure 5B, a clear separation between the groups was observed, indicating that the sPLS-DA model can accurately predict the differences between the G1, G2, and G3 groups.

**Figure 6:**
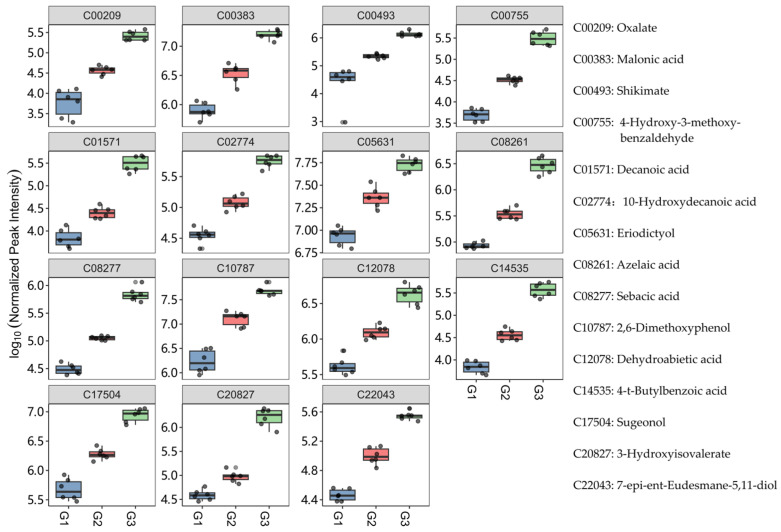
Trends of 15 differential metabolites in vivo.

We filtered for differential metabolites (DMs) at *p* < 0.01. As shown in Figure 5C,D, 77 DMs were filtered into CL4176 when it was treated by G2, of which 31 were upregulated and 46 were downregulated, and 60 DMs were upregulated and 3 DMs were downregulated in G3 compared with G2. Among these DMs, we observed one upregulated metabolite after G2 treatment that was downregulated in G3 and three downregulated metabolites that were upregulated in G3 (Figure 5E and Supplementary Figure S1). Meanwhile, after cleaning, we obtained 11 differential metabolites whose levels changed obviously after G3 treatment. Finally, we obtained 15 DMs in total, and their variation trends are shown in Figure 6.

**Figure 7:**
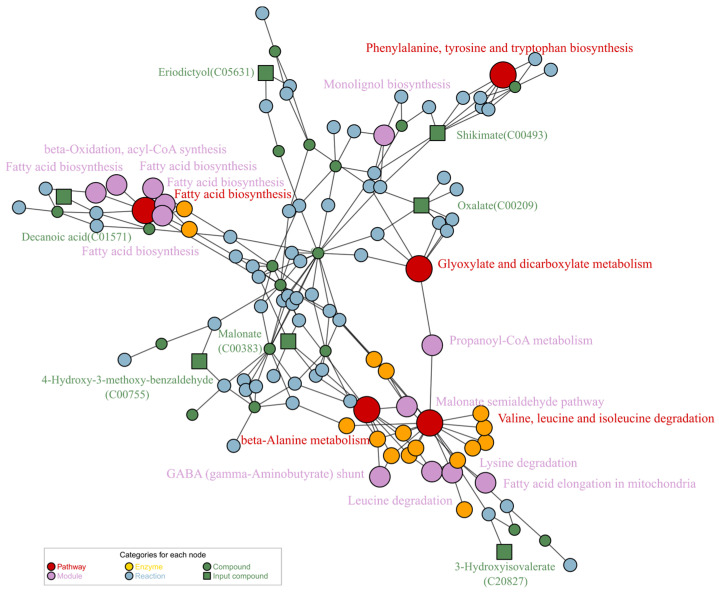
Mapped metabolic route network using FELLA enrichment. Node and label color represents element type: red, pathway; yellow, enzyme; green, compound; purple, module; blue, reaction. Green ○ represents related compounds, and green □ represents input DMs.

Fifteen DMs whose levels were significantly changed were functionally annotated by FELLA, and we selected the enriched KEGG nodes, using a *p* score < 0.01. Supplementary Table S2 shows the pathways and modules enriched in KEGG. The relationship among KEGG pathways, modules, enzymes, and reactions is shown in Figure 7. Five pathways that were affected after G2 and G3 treatments are fatty acid biosynthesis; valine, leucine, and isoleucine degradation; β-alanine metabolism; glyoxylate and dicarboxylic acid metabolism; and phenylalanine, tyrosine, and tryptophan biosynthesis. Additionally, 12 modules were also affected, including monolignol biosynthesis, the malonate semialdehyde pathway, propionyl-CoA metabolism (propanoyl-CoA => succinyl-CoA), fatty acid biosynthesis in mitochondria, fatty acid biosynthesis, beta-oxidation, acyl-CoA synthesis, fatty acid biosynthesis in mitochondria, GABA (gamma-aminobutyrate) shunt, fatty acid elongation in mitochondria, and leucine degradation.

### 3.6. Validation of Metabolic Regulatory Mechanisms by qRT-PCR

To confirm the degree of gene alterations in CL4176 in vivo after ScE treatment, we used an effective concentration of 50 μg/mL ScE. The metabolic pathways for the breakdown of valine, leucine, and isoleucine, as well as the creation of fatty acids, are connected to the metabolism of CL4176. Based on network relationship diagrams from the FALLA and KEGG enrichment analysis, we identified alanine–glyoxylate transaminase and enoyl-CoA hydratase as enzymes on these pathways. We discovered *T09B4.8* and *agxt-1* for alanine–glyoxylate transaminase and *ech-7* for enoyl-CoA hydratase through FALLA enrichment analysis. We used qRT-PCR to assess *T09B4.8*, *ech-7*, and *agxt-1* expression, which was reduced after ScE treatment.

**Figure 8:**
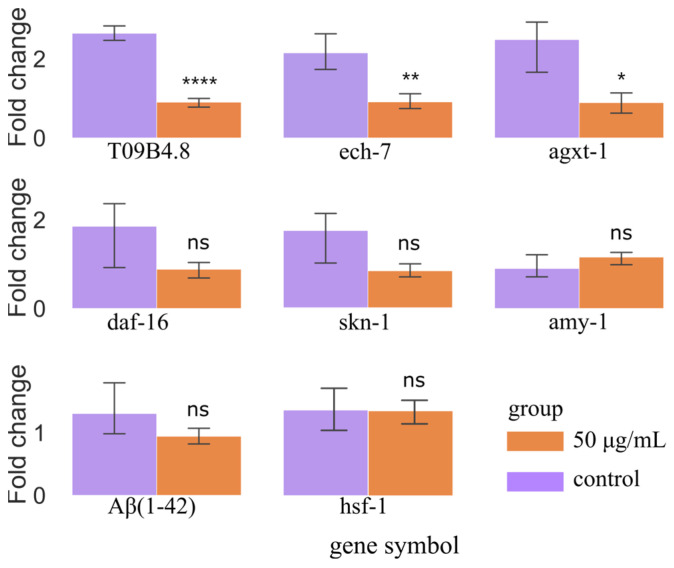
Validation of genes related to the regulation of ScE through qRT-PCR. *p* < 0.05 (*), *p* < 0.01 (**), *p* < 0.0001 (****), no significance (ns).

*C. elegans* skn-1, which plays an important role in regulatory pathways and treatments to increase life span, is significantly homologous to Nrf2, a regulatory protein for antioxidant and xeno defense [33]. Daf-16 and skn-1 play necessary roles in lowering Aβ aggregation, and daf-16 regulates the insulin/insulin-like growth factor receptor signaling system (IIS), which has been linked to transcriptional alterations in genes related to aging, development, stress, metabolism, and immunity [25]. Hsf-1 reduces Aβ proteotoxicity in the AD model of *C. elegans* [34]. We performed qRT-PCR to validate skn-1, DAF-16, and hsf-1 expression levels, which showed no significant difference between the ScE-treated group and the control group (Figure 8). We also used qRT-PCR on Aβ_1–42_ and amy-1 to determine whether ScE therapy affected their expression levels, as they are important biomarkers for AD development [27]. The findings demonstrated that there was no discernible difference in Aβ_1–42_ and amy-1 expression levels between the treatment and control groups. However, ScE treatment reduced the mean amount of Aβ that was deposited in the transgenic worm CL2006. Therefore, we propose that the prevention of ScE against Aβ aggregation might be the primary factor to relieve the paralytic behavior of the CL4176 strain.

## 4. Discussion

### 4.1. ScE Is a Highly Effective and Low-Toxicity Substance Group against Neurodegenerative Diseases

Various medicinal plants possess diverse medicinal values and are highly safe. They contain active components with anti-inflammatory, anti-amyloid, antioxidant, and autophagy-targeting properties, which could be used to create medicines against AD [35]. Sc is a medicinal plant with anti-inflammatory, antioxidant, and anticancer activities. Ethanol extract of Sc root can reduce acetylcholinesterase activity in AD rats [36]. The natural compounds (3,4-dihydroxybenzoic acid, catechin, and epicatechin) from Sc rhizomes have neuroprotective effects on rat cortical neurons with Aβ_25–35_ deposition [37]. Moreover, the n-BuOH extract of SC has antioxidant and acetylcholinesterase inhibitory properties [38]. AD is a neurodegenerative disease driven by aging, which affects the central nervous system irreversibly [39]. *C. elegans* has many advantages comparing rats or mice. For example, they have transparent bodies, which facilitate fluorescence observation and are easier to mass-produce. The transgenic worms of CL4176 displayed paralysis symptoms upon temperature induction, while *C. elegans* CL2006 displayed paralysis symptoms as the organism aged. In our investigation, ScE extended the life span of N2 *C. elegans*, suggesting that ScE has anti-aging effects. Based on these findings, we used transgenic worms and a metabolome analysis to explore the intrinsic connection between the inhibitory effect of Aβ and life span. In our subsequent research, ScE (1–100 μg/mL) showed dose-dependent anti-paralysis effects on the transgenic mutant CL4176. We found that ScE delayed paralysis of CL4176 obviously but did not extend the life span in 50 μg/mL. ScE (50 μg/mL) also reduced Aβ deposition significantly in the transgenic strain CL2006. In all effective concentrations, ScE had potential neuroprotective effects in AD development without obvious toxicity. In conclusion, we investigated the neuroprotective effects of ScE on the AD model of *C. elegans*. It was found that ScE not only prolonged the life span of healthy *C. elegans* and the paralysis time of AD model worms but also reduced the Aβ deposition in the AD model of *C. elegans.*

Additionally, we performed an LC-HRMS analysis on ScE and discovered that the alkaloids compound nicotinamide (PubChem CID 936) and carbohydrates compound uridine 5′-monophosphate (PubChem ID 6030) were relatively high in addition to the most relatively abundant compounds. Niacinamide can improve neuronal survival and alleviate neuronal cell death in developing rats [40]. Uridine 5′-monophosphate is a uridine cycle precursor. In vivo, uridine increases neurite formation in PC-12 cells. A combination of uridine and docosahexaenoic acid improves neurocognitive impairments [41]. Given the limited number of compounds identified in our database, we believe that nicotinamide (CID 936 in Figure 1) and uridine-5′-monophosphate (CID 6030 in Figure 1) may be two of the active ingredients that are responsible for the neuroprotective effects of ScE.

### 4.2. Metabolic Pathways Affected by ScE in CL4176

Metabolomics is crucial as a high-throughput screening technique for illuminating the pathogenesis of AD since it enables the simultaneous detection and quantification of multiple disturbed metabolites in tissues or biological fluids and replicates the metabolic reaction networks affected by the disease [42]. According to our metabolomic analyses, multiple metabolic pathways and modules were disrupted in CL4176 after ScE treatment (Figure 7). Clinical research has demonstrated that the levels of BCAAs (valine, leucine, and isoleucine) are related to AD, and the deficiencies and accumulation in BCAAs can cause tau protein phosphorylation and the development of AD [43]. Low concentrations of serum BCAAs can increase the risk of AD [44]. In the blood of adults, BCAAs metabolism and synthesis pathways have a dual influence on aging. Leucine and isoleucine levels decrease with aging, whereas valine levels increase in the aging process [45]. Our pathway enrichment disclosed related pathways of valine, leucine, and isoleucine degradation (*p* < 0.0001). Leucine degradation (M00036) (*p* < 0.01), which is involved in valine, leucine, and isoleucine degradation pathways, also had significant effect values. Carnosine is potentially protective against Aβ-induced cytotoxicity in vivo [46]. Anserine is a methylated derivate of carnosine; it can improve memory function in AD model mice. β-alanine is a metabolite of carnosine/anserine, and increased serum β-alanine levels reduce the incidence of AD [47]. The malonate semialdehyde pathway (M00013) (*p* < 0.001) is involved in β-alanine metabolism and propionic acid metabolism, which is linked to the deregulation of certain miRNAs in the AD model *Drosophila* [48]. In our investigation, the β-alanine metabolic pathway was also affected. According to KEGG entities, γ-aminobutyrate (GABA) shunt (M00027) (*p* < 0.01) was associated with alanine, aspartate, and glutamate metabolism. A metabolomic analysis of AD subjects revealed that increased alanine levels were found in the brain gray matter, while lower aspartate levels were found in the brain white matter. AD participants also had higher amounts of glutamate, and neuronal death was linked to the dysregulation of the glutamatergic system [49]. Changes in glutamate metabolism can impact GABA metabolism because glutamate decarboxylase converts glutamate to GABA, which can be derived from GABA [50].

Unsaturated fatty acids (MUFAs), polyunsaturated fatty acids (PUFAs), and saturated fatty acids (SAFAs) are different types of fatty acids that are components of phospholipids [51]. Fatty acid imbalances can be a sign of neurological and other mental diseases; elevated levels of SAFAs and MUFAs and decreased levels of PUFAs in the brain tissue are found in AD patients [52]. A metabolomic study of brain tissue and blood showed that fatty acid biosynthesis is disrupted in clinical AD [21]. Dietary lipids slow down aging and prolong the life span of *C. elegans* by the unsaturated fatty acid biosynthesis pathway [53]. In β-oxidation, acyl-CoA synthesis (M00086) (*p* < 0.01) is associated with fatty acid biosynthesis and degradation. The fatty acid biosynthesis (*p* < 0.001) pathway was disturbed in our pathway analysis. In addition, we discovered that, after ScE treatment, CL4176 altered glyoxylate and dicarboxylate metabolism, as well as the phenylalanine, tyrosine, and tryptophan pathway. These findings demonstrate the modulation of metabolic pathways in CL4176.

From our metabolites’ enrichment, we also found that 4-hydroxy-3-methoxybenzaldehyde (C00755), decanoic acid (C01571), and eriodictyol (C05631) and others of CL4176 were significantly affected after treatment with ScE exposure. Vanillin is a class of phenolic compounds with antioxidant potential that improves cognitive function and memory impairment in AD rats [54]. The metabolite 4-hydroxy-3-methoxy-benzaldehyde is a deuterated form of vanillin. When applied in mice, eriodictyol, a naturally occurring flavonoid with anti-inflammatory, antioxidant, and anticancer properties, lessens neuroinflammation and cognitive deficits [55]. Moreover, exogenous eriodictyol helps AD mice with their cognitive deficits [56]. Decanoic acid, a medium-chain fatty acid, reduces oxidative stress and enhances neuronal health by boosting catalase activity in neuronal cells [57]. We found that decanoic acid, 4-hydroxy-3-methoxybenzaldehyde, and eriodictyol levels were obviously higher following ScE treatment. This also supported the possibility that ScE has neuroprotective properties.

### 4.3. Regulatory Sites of ScE Repairing AD Metabolic Disorders

In our research, fatty acid biosynthesis and valine, leucine, and isoleucine degradation pathways may be the primary mechanisms through which ScE delivers its neuroprotective effects. We then combined KEGG pathway enrichment and GO analysis to yield the genes associated with regulatory sites of ScE. According to GO and NCBI, ech-7 is directly homologous to human *ECHS1*. *ESHS1* encodes the short-chain enoyl-CoA hydratase (SCEH), which is involved in fatty acid β-oxidation in mitochondria and catabolism of valine in the nervous system [58]. *ECHS1* deficiency causes dyskinesia in valine metabolic disorders or leads to severe metabolic disorders, such as Leigh-like syndrome [59,60]. The gene ech-7 affects decanoic acid (C01571) production by regulating acetyl-CoA carboxylase. Decanoic acid is the end product of the fatty acid biosynthesis pathway. Decanoic acid can improve cognitive disorders and decreases the expression of Aβ_42_ in the brain tissues of AD mice [61]. In our study, the decanoic acid level increased significantly with ScE exposure, suggesting that ScE may have an effect on CL4176 by upregulating decanoic acid levels.

The metabolite 3-hydroxyisovalerate (C20827) is an intermediate on the valine, leucine, and isoleucine degradation pathway. The genes *T09B4.8* and *agxt-1* regulate alanine–glyoxylate transaminase, then affecting 3-hydroxyisovalerate. Based on NCBI, *T09B4.8* is similar to human *AGXT2*, and mutations in *AGXT2* may contribute to the development of atrial fibrillation and age-related thromboembolic complications [62]. *Agxt-1* is orthologous to human *AGXT*; the *AGXT* gene mutation can cause primary hyperoxaluria type I in rats [63]. Metabolite 3-hydroxyisovalerate is lower in AD patients than in controls [64]. We discovered that the level of 3-hydroxyisovalerate considerably rose following ScE treatment, indicating that ScE may exhibit neuroprotective effects via increasing the amount of 3-hydroxyisovarate through *T09B4.8* and *agxt-1*. Our investigation revealed obvious changes in the levels of *ech-7*, *T09B4.8*, and *agxt-1* genes, which are related to acetyl-CoA carboxylase and alanine–glyoxylate transaminase. ScE may play a metabolic regulatory role by regulating the expression levels of *ech-7*, *T09B4.8*, and *agxt-1* through the fatty acid biosynthesis and valine, leucine, and isoleucine degradation pathways. Since we do not fully understand the functions of these genes, we are unable to determine whether they are important genes that exert neuroprotection.

## 5. Conclusions

In conclusion, we investigated the neuroprotective effects of ScE on an AD model of *C. elegans.* It was found that ScE not only prolonged the life span of healthy *C. elegans*s and the paralysis time of CL4176 but also reduced Aβ deposition in CL2006. Through phenotypic investigation, metabolite analysis, and qRT-PCR validation, we discovered that the neuroprotective effects of ScE were associated with the degradation of valine, leucine, and isoleucine, as well as fatty acid biosynthesis. We also first found that the gene expression levels of *T09B4.8*, *agxt-1*, and *ech-7* were associated with neuroprotection. Our research provides theoretical support for the mechanism of ScE neuroprotection through metabolic regulation and offers novel insights into the development of natural plants with therapeutic efficacy in treating AD through functional foods.

## Data Availability

The data presented in this study are available in the article and Supplementary Materials.

## Supplementary Materials

The following supporting information can be downloaded at https://www.mdpi.com/article/10.3390/metabo14010049/s1, Figure S1: Chromatogram of ScE; Figure S2: SCE’s impact on the body length of *C. elegans*; Table S1: Putatively annotated ScE compounds by LC-HRMS; Table S2: Enrichments of differential metabolites invoked by SCE (G3/G2 and G2/G1); Table S3: Primers used for qRT-PCR analysis; Table in the supplementary xlsx file: Metabolic data of CL 4176 (G1, G2, G3).
