# Supplementary material for: Metabolic Regulations of Smilax china L. against β-Amyloid Toxicity in Caenorhabditis elegans
Source: Metabolites. 2024 Jan 13;14(1):49. doi: 10.3390/metabo14010049 (PMC10818737; doi:10.3390/metabo14010049)
Supplement: Supplementary file 1 [file metabolites-14-00049-s001.zip › Supplementary information.pdf]

Supplementary:

Metabolic Regulations of *Smilax china* L. against β-Amyloid Toxicity in *Caenorhabditis elegans*

Lili Yan [1], Yuchan Deng [1], Yulan Du [1], Xutong Fang [1], Xin Fang [2,*] and Qiang Zhang [1,*]

1 Shaanxi Key Laboratory of Natural Products & Chemical Biology, College of Chemistry & Pharmacy, Northwest A&F University, Yangling 712100, China

2 State Key Laboratory of Phytochemistry and Plant Resources in West China, Kunming Institute of Botany, Chinese Academy of Sciences, Kunming 650201, China

* Correspondence: xinfang@mail.kib.ac.cn (X.F.); zhangq@mail.nwsuaf.edu.cn (Q.Z.)

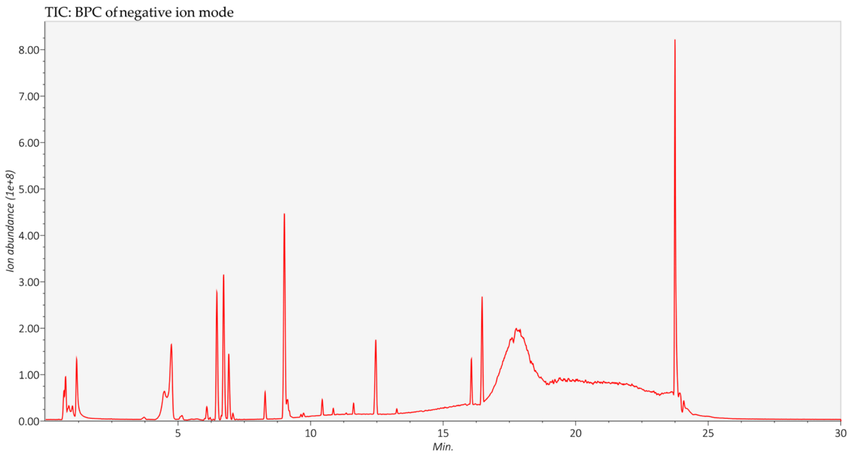

(1). Base peak chromatogram of negative ion mode.

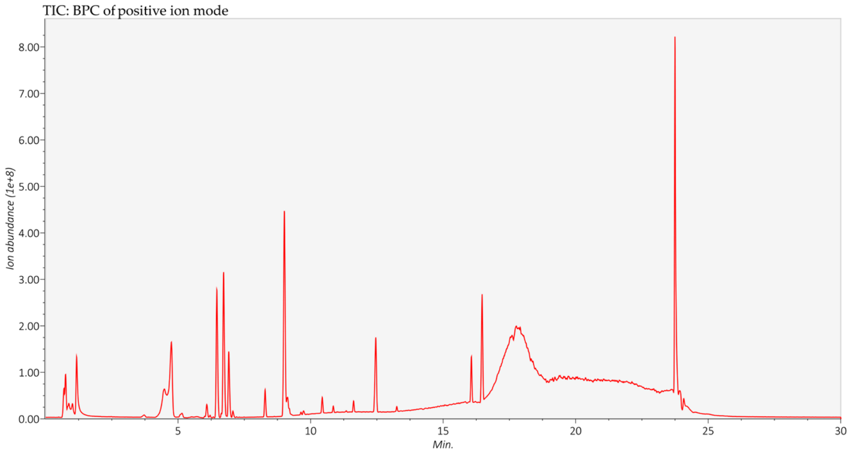

(2). Base peak chromatogram of positive ion mode.

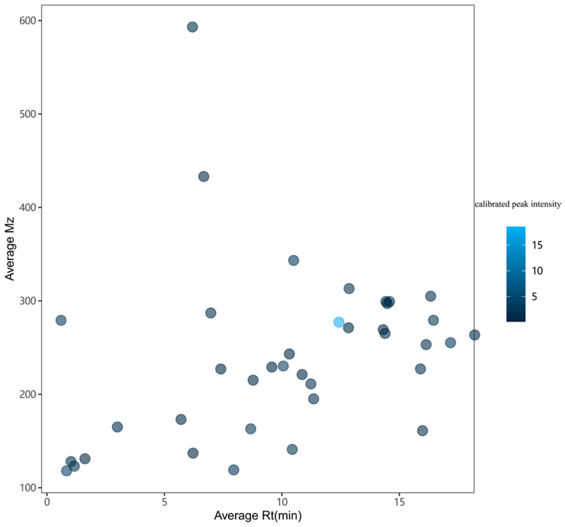

(3). Two-dimensional chromatograms of SCE.

Figure S1. Chromatogram of SCE.

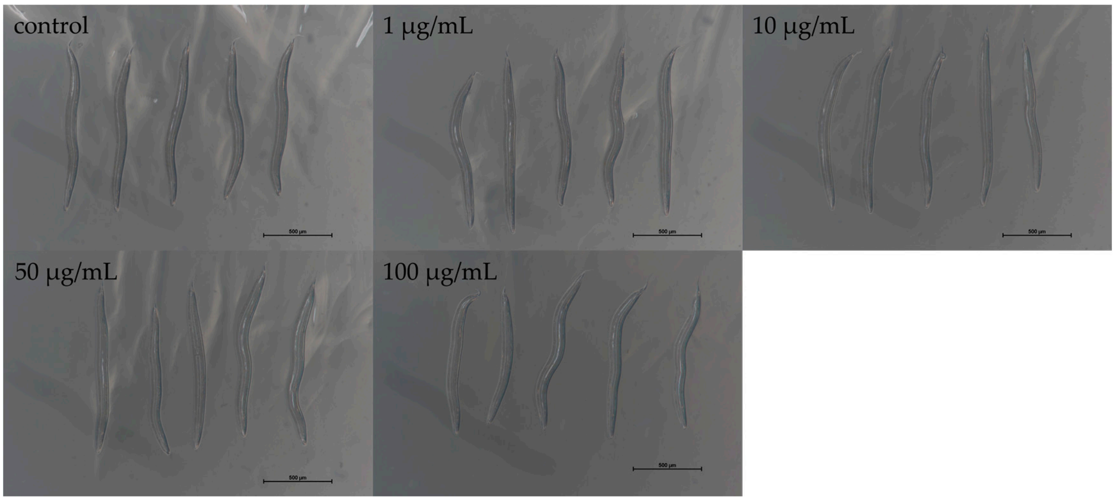

Selected pictures of *C. elegans* with ScE treatment. (30 worms per treatment)

Figure S2. SCE's impact on the body length of *C. elegans.*

Table S1. Putatively annotated ScE compounds by LC-HRMS.

PubChem CID	RT (min)	MF	Mode	Adduct	Average M/Z	MS/MS spectrum
162935541	12.429	$C_{17}H_{26}O_3$	ESI-	[M-H]-	277.18063	183.89915,6717 277.18076,2628972
1182	0.836	$C_5H_{11}NO_2$	ESI+	[M+H]+	118.08617	58.02928,101665 70.06555,61325 72.08114,2314811 76.93327,76405 87.53455,29869 109.06461,15421 118.0861,4133996
15433	10.061	$C_{14}H_{31}NO$	ESI+	[M+H]+	230.24706	57.07036,223078 61.7001,7323 62.06047,251437 69.07024,48264 93.06991,24477 109.10109,116048 169.98953,14615 176.15083,203862 187.97575,16233 211.18799,11106 230.24692,4475846
985	17.181	$C_{16}H_{32}O_2$	ESI-	[M-H]-	255.23265	71.00635,7920 98.13085,7998 116.92714,11256 170.98654,32485 227.79988,9826 254.98607,55619 255.23274,5396950
3026	0.599	$C_{16}H_{22}O_4$	ESI+	[M+H]+	279.15814	92.65131,7284 132.95798,17531 149.02284,3662536 165.54724,7138 195.08026,14813 218.93042,17679 226.93771,8929 275.90082,23268
20281	10.502	$C_{19}H_{39}N_2O_3$	ESI+	[M]+	343.29437	53.46519,4336 93.61445,4573 123.11646,5846 183.17366,104428 240.23119,5162172 297.06372,16958 320.91818,25488 343.29407,348967
12500	7.949	C_9H_{10}	ES+	[M+H]+	119.08543	76.9332,10188 79.0215,18822 91.05434,1234786 103.05422,34330 117.06971,79500 119.08529,1868532
936	1.165	$C_6H_6N_2O$	ESI+	[M+H]+	123.05508	62.92944,13069 76.5154,22949 80.04971,308599 88.0043,8684 90.94786,6819 105.04462,24939 123.055,3279426
10146	10.441	$C_{19}H_{21}NO_2$	ESI-	[M-H]-	141.01581	59.01258,4164605 92.99452,15529
5281426	8.675	C9H6O3	ESI+	[M+H]+	163.03848	58.99792,35443 80.94524,9327 95.04919,66698 117.06968,47634 123.03999,178999 133.02798,266704 141.05029,67880

163.03835,2543588

PubChem CID	RT (min)	MF	Mode	Adduct	Average M/Z	MS/MS spectrum
5280450	16.454	$C_{18}H_{32}O_2$	ESI-	[M-H]-	279.23267	59.01251,8428 192.98798,8116 238.99348,16070 258.99936,15203 278.18475,18862 279.23276,4211488
15385367	11.236	$C_{12}H_{20}O_3$	ESI-	[M-H]-	211.13329	59.01257,8527 104.99465,4777 116.99471,3817 122.03176,13283 139.03902,10554 151.91724,4867 156.96126,27123 190.99217,17558 211.13333,432235
66556493	11.352	$C_{12}H_{20}O_2$	ESI-	[M-H]-	195.13823	101.15147,3915 167.1432,90723 175.13251,4068 195.13828,2327946
662	6.975	$C_{15}H_{12}O_6$	ESI-	[M-H]-	287.056	57.03337,13972 83.01253,21619 123.04411,3395 125.02326,414329 137.02351,2954 169.01315,2927 177.05481,39210 199.07581,14117 215.07091,27748 227.97272,11434 243.06598,87223 259.06107,583874 287.05612,678430
499	1.024	$C_5H_7NO_3$	ESI-	[M-H]-	128.03418	55.75593,4805 74.80853,5232 94.66902,5662 128.03423,936603
10358150	12.862	$C_{19}H_{20}O_4$	ESI+	[M+H]+	313.1398	57.07027,10795 81.07004,5651 115.05382,6060 133.10074,3716 163.93968,2793 233.6207,3300 253.11876,191063 313.13959,558069
6030	16.335	$C_9H_{13}N_2O_9P$	ESI-	[M-H2O-H]-	305.02286	59.01257,425324 125.45128,4210 141.01581,1026044 201.01418,9976 223.01933,37077 244.98425,19027 304.98355,34015
8205	15.9	$C_{14}H_{28}O_2$	ESI-	[M-H]-	227.20108	59.01248,4331 80.62833,1595 125.92764,19383 140.99454,3221 142.99144,44374 162.9977,31304 186.99818,15493 211.13351,3527 227.2011,923234
57404492	16.141	$C_{16}H_{30}O_2$	ESI-	[M-H]-	253.21696	61.98714,4523 83.04889,12305 130.94859,4981 204.98868,10337 235.20592,12656 253.21703,2634709
11833	15.988	$C_{10}H_8O_2$	ESI-	[M+H]+	161.05928	72.0448,2577 81.04495,3762 93.04504,5871 103.05424,19140

						105.06992,22483 120.05547,69449 121.06343,53133 133.06445,390129 161.0592,444326
625291	14.584	$C_{20}H_{28}O_2$	ESI-	[M-H]-	299.20148	55.60971,1738 166.99162,3660 174.99158,8255 210.99857,2724 259.00046,8830 279.00568,12730 299.20151,1315994
445639	17.361	$C_{18}H_{34}O_2$	ESI-	[M-H]-	281.24838	52.9985,2256 103.97529,5542 116.92733,5480 190.99271,3282 220.98416,4395 239.58968,2924 243.00493,3520 281.24847,1424063
14921167	14.489	$C_{16}H_{26}O_3S$	ESI-	[M-H]-	297.15274	59.01244,4329 170.00307,5796 183.01115,159681 256.98383,14154 296.23114,13620 297.15283,2491900

PubChem CID	RT (min)	MF	Mode	Adduct	Average M/Z	MS/MS spectrum
5483905	6.196	$C_{27}H_{30}O_{15}$	ESI-	[M-H]-	593.15149	100.74881,1234 123.04398,4364 137.02338,2614 171.04398,2613 189.05505,2338 219.03038,1875 241.05014,4770 255.02965,65896 284.03256,370886 511.34961,7636 593.15167,1002560
1017	2.999	$C_8H_6O_4$	ESI-	[M-H]-	165.01836	68.99432,993 121.02832,726688 145.00964,1090 164.03445,989 165.01839,67606
23628197	10.862	$C_{14}H_{22}O_2$	ESI-	[M-H]-	221.15405	54.39076,3335 92.99438,3509 103.91888,13160 192.98846,6271 205.12289,11354 221.15416,1590646
8778	14.394	$C_{12}H_{26}O_4S$	ESI-	[M-H]-	265.14777	61.98705,3992 78.95777,12732 96.95882,289522 108.98974,1711 178.99171,5550 224.99763,7169 265.14774,879940
439885	14.428	$C_{18}H_{36}O_3$	ESI-	[M-H]-	299.259	56.70603,2848 116.9275,5534 174.99159,9770 210.99901,4354 238.99379,8185 253.25342,13066 281.24832,18356 299.259,1526379
10457	5.707	$C_8H_{14}O_4$	ESI-	[M-H]-	173.08102	59.01259,8625 72.99183,6639 83.04661,1366 99.92458,3253 111.08033,285440 123.94518,1239 129.09099,23418 146.02371,1220 172.82965,6273 173.08102,190793
5282997	14.313	$C_{16}H_{30}O_3$	ESI-	[M-H]-	269.2121	59.01262,7887 81.03332,3332 164.99588,6693 182.98697,4698 204.98875,5787 220.98369,7049 251.20137,34706 269.21213,622896
21776027	12.837	$C_{16}H_{32}O_3$	ESI-	[M-H]-	271.22781	116.99464,1292 154.99185,1666 186.99796,8090 190.99159,2632

						210.99858,6812 225.22171,2844 242.17572,2386 270.21613,8233 271.22775,698270
15816	8.776	$C_{11}H_{20}O_4$	ESI-	[M-H]-	215.12828	57.03333,1574 63.97582,818 116.92741,3700 125.87215,1863 152.99585,886 166.99152,15219 171.13794,2214 174.9915,8503 186.99782,3525 197.11752,125414 214.99313,38702 215.12823,330223
10458	10.319	$C_{13}H_{24}O_4$	ESI-	[M-H]-	243.15987	85.59893,1496 152.99593,3268 181.15889,102441 194.98694,12621 214.99355,3458 225.14911,224130 243.15982,562613
92794	6.676	$C_{21}H_{22}O_{10}$	ESI-	[M-H]-	433.1138	59.01257,16780 93.03329,16637 107.01266,7095 145.02834,2566 151.00261,131811 177.01845,14557 244.9754,5536 269.0455,23082 271.06116,730708 414.94269,2483

PubChem CID	RT (min)	MF	Mode	Adduct	Average M/Z	MS/MS spectrum
445154	7.399	$C_{14}H_{12}O_3$	ESI-	[M-H]-	227.07094	139.11169,6285 142.99152,17028 154.94551,2706 159.08063,6391 172.95636,7409 182.07268,3701 185.06003,53025 207.00491,10269 225.0551,2747 227.07092,529512
12736	9.572	$C_{12}H_{22}O_4$	ESI-	[M-H]-	229.14406	53.56525,812 92.99445,9905 103.93913,1072 129.05461,5212 140.99472,6369 156.91995,1771 161.00096,7887 173.92519,3083 181.00728,6364 208.98404,11753 211.1333,138895 229.14406,349156
338	6.217	$C_7H_6O_3$	ESI-	[M-H]-	137.02332	67.02889,1168 93.0333,245268 105.95905,1398 118.02854,1015 136.86176,17681
10349	1.619	$C_5H_8O_4$	ESI-	[M-H]-	131.03387	59.0126,12819 72.99184,8715 85.06455,5883 87.04388,167435 112.03951,624 131.03387,49501

CID, pubchem cid. All the structures can be acquired directly from PubChem (https://pubchem.ncbi.nlm.nih.gov) by CID.

Table S2. Enrichments of differential metabolites invoked by SCE (G3/G2 and G2/G1).

KEGG ID	KEGG name	*p* value
cel00061	Fatty acid biosynthesis	0.00104608
cel00280	Valine, leucine and isoleucine degradation	0.000001
cel00410	beta-Alanine metabolism	0.0001506
cel00630	Glyoxylate and dicarboxylate metabolism	0.00285858
cel00400	Phenylalanine, tyrosine and tryptophan biosynthesis	0.00806879
M00039	Monolignol biosynthesis, phenylalanine/tyrosine => monolignol	3.3978E-05
M00013	Malonate semialdehyde pathway, propanoyl-CoA => acetyl-CoA	0.0009316
M00741	Propanoyl-CoA metabolism, propanoyl-CoA => succinyl-CoA	0.0012695
M00874	Fatty acid biosynthesis in mitochondria, fungi	0.00472245
M00082	Fatty acid biosynthesis, initiation	0.00635424
M00086	beta-Oxidation, acyl-CoA synthesis	0.00766395
M00873	Fatty acid biosynthesis in mitochondria, animals	0.00739053
M00027	GABA (gamma-Aminobutyrate) shunt	0.00860417
M00083	Fatty acid biosynthesis, elongation	0.01226873
M00085	Fatty acid elongation in mitochondria	0.0187349
M00032	Lysine degradation, lysine => saccharopine => acetoacetyl-CoA	0.04689724
M00036	Leucine degradation, leucine => acetoacetate + acetyl-CoA	0.01178182

Table S3. Primers used for qRT-PCR analysis.

NCBI Gene ID	Cene name	Oligo Name	sequence (5′ to 3′)
172981	DAF-16	DAF-16-F	TACATTGCTCGAAGTGCCGA
		DAF-16-R	TCAGATGGTAGCGGCGAATC
177343	skn-1	skn-1-F	TCTACTCTTTCCTCCCTTCGG
		skn-1-R	ACAAGGGGATGGGGATCAGA
173078	hsf-1	hsf-1-F	AGGAGGACCATCTACATCATCTTCG
		hsf-1-R	CTATTCACCTGAGCCATTTGCCTAG
172301	T09B4.8 R	T09B4.8-F	TCAGTTGGACATTGTCATCCGTGAG
		T09B4.8-R	GCTTCTGTGCTTGCTCGGTAAGAG
850514	agx-1	agxt-1-F	TGACCGATCTCTGGGTGTCTTCTC
		agx-1-R	GCGGCAAATATCTCTGTGACTCCTC
173300	ech-7	ech-7	AGGCTGTTCTGTTGGCTGATAGG
		ech-7-R	AGGTAAGCAGGCGTAGGCATTC
	Amy-1	Amy-1-F	CCGACATGACTCAGGATATGAAGT
		Amy-1-R	CACCATGAGTCCAATGATTGCA
	Aβ(1-42)）	Aβ(1-42)-F	CCGACATGACTCAGGATATGAAGT
		Aβ(1-42)-R	CACCATGAGTCCAATGATTGCA
	Ref	β-actin-F	ATCCATCGTTCACCGCAAGT
		β-actin-R	TAAGGACAAAAATGGGGCGG
